# Prevention of occupational hand eczema in healthcare workers during the COVID‐19 pandemic: A controlled intervention study

**DOI:** 10.1111/cod.14206

**Published:** 2022-08-30

**Authors:** Cara Symanzik, Lukasz Stasielowicz, Richard Brans, Christoph Skudlik, Swen Malte John

**Affiliations:** ^1^ Department of Dermatology, Environmental Medicine and Health Theory Osnabrück University Osnabrück Germany; ^2^ Institute for Interdisciplinary Dermatological Prevention and Rehabilitation (iDerm) Osnabrück University Osnabrück Germany; ^3^ Institute of Psychology Osnabrück University Osnabrück Germany

**Keywords:** COVID‐19, education, hand eczema, healthcare, hygiene, intervention, occupational, pandemic, prevention, skin care

## Abstract

**Background:**

Occupational hand eczema (HE) is common among healthcare workers (HCWs) and has—in some regions of the world—increased during the COVID‐19 pandemic due to related hygiene measures.

**Objectives:**

To evaluate the efficacy of an intervention for HE prevention in HCWs during the pandemic.

**Methods:**

A prospective, controlled, unblinded interventional trial was conducted in 302 HCWs. The intervention group (IG) (*n* = 135) received online‐based health education and free access to hand cleansing and hand care products. The control group (CG) (*n* = 167) did not receive any intervention within the study. At baseline (T0), after 3 (T1) and 6 (T2) months, participants completed standardized questionnaires. The Osnabrueck Hand Eczema Severity Index (OHSI) was assessed at T0 and T2.

**Results:**

During the observation period, there were no new HE cases in the IG (*n* = 115) and 12 cases (8.8%) in the CG (*n* = 136). OHSI values at T2 were lower in the IG (*b* = −1.44, *p* < 0.001). Daily use of emollients was higher at work (*b* = 1.73, *p* < 0.001) and at home (*b* = 1.62, *p* < 0.001) in the IG at T2.

**Conclusions:**

The intervention was effective in HE prevention and improving skin care behaviour during the COVID‐19 pandemic.

## Graphical abstract

The two‐part intervention (free access to an adequate hand cleansing product and emollient plus free access to eHealth education) was effective not only in prevention of incident hand eczema but also in improving the skin condition of the hands. The described intervention can be used as is in healthcare workers.

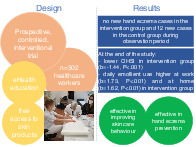

## 1 INTRODUCTION

Healthcare workers (HCWs) are exposed to a considerable amount of wet work^1^ and thus, at high risk of developing hand eczema (HE), mainly caused by irritant contact dermatitis.^2^ A 1‐year prevalence of HE in HCWs of around 20% has been reported previously.^2^ Occupational HE is often chronic, burdensome and associated with impaired quality of life.^3^, ^4^, ^5^ In some cases, it may even result in leaving the workforce. Consequently, the health economic burden is high due to direct (e.g., medical treatment costs) and indirect costs (e.g., costs for sickness‐related work absences).^6^, ^7^

Intensified hand hygiene measures have been implemented for containing the coronavirus disease 2019 (COVID‐19) pandemic^8^ which emerged in the beginning of 2020 and has led to increased skin strain in the general population and particularly in HCWs who face a double burden due to elevated hygiene measures both in private life and at work.^9^, ^10^ Accordingly, recent studies from different countries have demonstrated that prevalence of occupational dermatoses in HCWs has increased during the COVID‐19 pandemic.^11^, ^12^, ^13^ In our study period, more than 100 000 occupational COVID‐19 infections in HCWs were reported in Germany leading to further tightening of hygiene measures and an unprecedentedly high incidence of contact dermatitis.

In several intervention studies, it has been shown that health education is effective in the prevention of occupational hand eczema.^14^, ^15^, ^16^ Recommendations for preventing occupational HE include the use of adequate skin cleansing substances and skin care products,^17^ which can be conveyed by health education. This study aims at evaluating the efficacy of a complex two‐part intervention (online‐based health education plus provision of adequate skin products) in HCWs with respect to prevention of incident hand eczema and improvement of existing skin changes of the hands. Moreover, skin care behaviour (i.e., frequency of emollient use) and factors associated with incident hand eczema were investigated.

## 2 MATERIALS AND METHODS

### 2.1 Trial design

This study was a monocentric, prospective, interventional, controlled trial conducted in two hospitals in Lower Saxony, Germany. The study was conducted in agreement with the principles expressed in the Declaration of Helsinki. Ethic approval was obtained by the sub‐commission on the evaluation of medical research involving human subjects at the Medical Chamber of Lower Saxony, Hannover, Germany (procedure number 30/34/2020). The trial was prospectively registered at the German Clinical Trials Register (DRKS) (number DRKS00022957).

### 2.2 Participants

HCWs from 35 wards were recruited in two hospitals in Osnabrück and in Bad Rothenfelde which are located 30 km away from each other in Lower Saxony, Germany. After gaining permission from the hospitals' hygiene managements, nursing directorates, staff councils, medical directorates and company health managements, volunteers of one hospital (Osnabrück) were allocated to the intervention group (IG) and volunteers of the other hospital (Bad Rothenfelde) were allocated to the control group (CG). No further randomization within hospitals was done to avoid cross‐contamination among the participants in terms of knowledge and study products. The inclusion criteria were written informed consent, being of legal age and working in healthcare (e.g., nurses, surgical assistants, physiotherapists). HCWs with known allergies against fragrances and/or oat flour could not participate in the IG due to the composition of the provided study products. Termination criteria were adverse skin reactions from the study products (only IG) or discontinuation of working in healthcare.

### 2.3 Intervention group

The two‐part intervention comprised free access to a lipid‐containing syndet and an emollient for use both at work and at home accompanied by free access to an online training course on the prevention of hand eczema consisting of an e‐learning video of 35‐min length. An asynchronous store‐and‐forward technology was chosen to enable flexible access to the educational intervention and to avoid face‐to‐face education during the COVID‐19 pandemic. Schedule planning for the online‐based health education with the indicative target ‘Participants implement a considerate and careful skin cleansing and skin care behaviour within their private and occupational surroundings’ is provided in Table S1.

After recruitment and baseline data collection, participants received a handout with the key information about the online‐based health education which included a link and a quick response (QR) code with which the video could be retrieved. Furthermore, participants were provided with an information leaflet about HE, including a link list with additional helpful short videos about skin cleansing and skin care (Appendix S2), and additional information sheets about the appropriate use of protective gloves and adequate skin care behaviour. Participants initially received a starter kit containing four packages of the lipid‐containing syndet and four packages of the emollient. Further packages could be demanded by the participants without limitation over the whole observation period. The ingredients of the study products are listed in Table S2.

### 2.4 Control group

The CG did initially not receive an intervention within the study. No changes were made to the access to skin products provided by the hospital (treatment as usual). After the study was completed, the CG received access to the online‐based health education and was provided with one package each of the lipid‐containing syndet and the emollient.

### 2.5 Outcomes and assessment instruments

The primary outcome was presence/absence of HE, which was primarily assessed by using the validated Osnabrueck Hand Eczema Severity Index (OHSI)^18^, ^19^, ^20^ at T0 (baseline) and T2 (after 6 months). All skin examinations in the CG were conducted by a dermatologist experienced in occupational skin diseases (S.M.J.). The same dermatologist performed the (unblinded) skin examination in the IG, partially assisted by two other experienced occupational dermatologists. HE was defined as the presence of (i) vesicles or (ii) erythema score >2 in combination with a score >2 for at least one of the following clinical signs on the hands (papules, scaling, fissures) based on the OHSI assessment (modified from Reich et al.^21^). The primary outcome was additionally assessed by using a paper‐based questionnaire, including the question: ‘Do you currently suffer from hand eczema’ as well as additional questions about atopy (‘Have you ever had an itchy rash that has been coming and going for at least 6 months, and at some time has affected skin creases?’), which was designed in consideration of the Nordic Occupational Skin Questionnaire (NOSQ‐2002)^22^ and distributed at T0, T1 (after 3 months) and T2. Individual HE signs assessed by the OHSI and the overall OHSI score were considered in terms of a secondary outcome. Other secondary outcomes were skin care behaviour (i.e., frequency of emollient use at work and at home) which were assessed by using the aforementioned questionnaire at T0, T1 and T2.

### 2.6 Statistical methods

Data were analysed in the sense of an intention‐to‐treat analysis. Multiple imputation (30 imputations) was conducted by applying fully conditional specification^23^ to counterbalance missing data. In terms of descriptive statistics, frequencies and percentages were calculated for categorical variables and mean ± standard deviation (SD) for continuous variables using IBM SPSS Statistics (version 26). Inferential analyses were performed using R (version 4.0.2). The level of significance was *p* < 0.05. With respect to the primary and secondary outcomes, linear or logistic multilevel modelling (MLM) was used (Tables S3 and S4). Full R code and output is available at osf (https://osf.io/tyshu/?view_only=dca9217a95d743b1ac8c2cc3fcacda6c). For examining factors possibly related to developing HE in the CG, logistic regression was conducted by using OHSI‐based assessment data at T2 as dependent variable, and several baseline variables (e.g., atopy) as predictors.

## 3 RESULTS

### 3.1 Recruitment

Recruitment started on 1 December 2020 and was finished on 29 January 2021. This recruitment interval was necessary due to organizational reasons (e.g., work schedules, holidays etc.). A total of 302 HCWs were included in this study. Follow‐ups were done in 3‐month intervals between T0 and T1 as well as T1 and T2 (i.e., 6‐month observation period overall for each participant).

### 3.2 Participant flow

**FIGURE 1:**
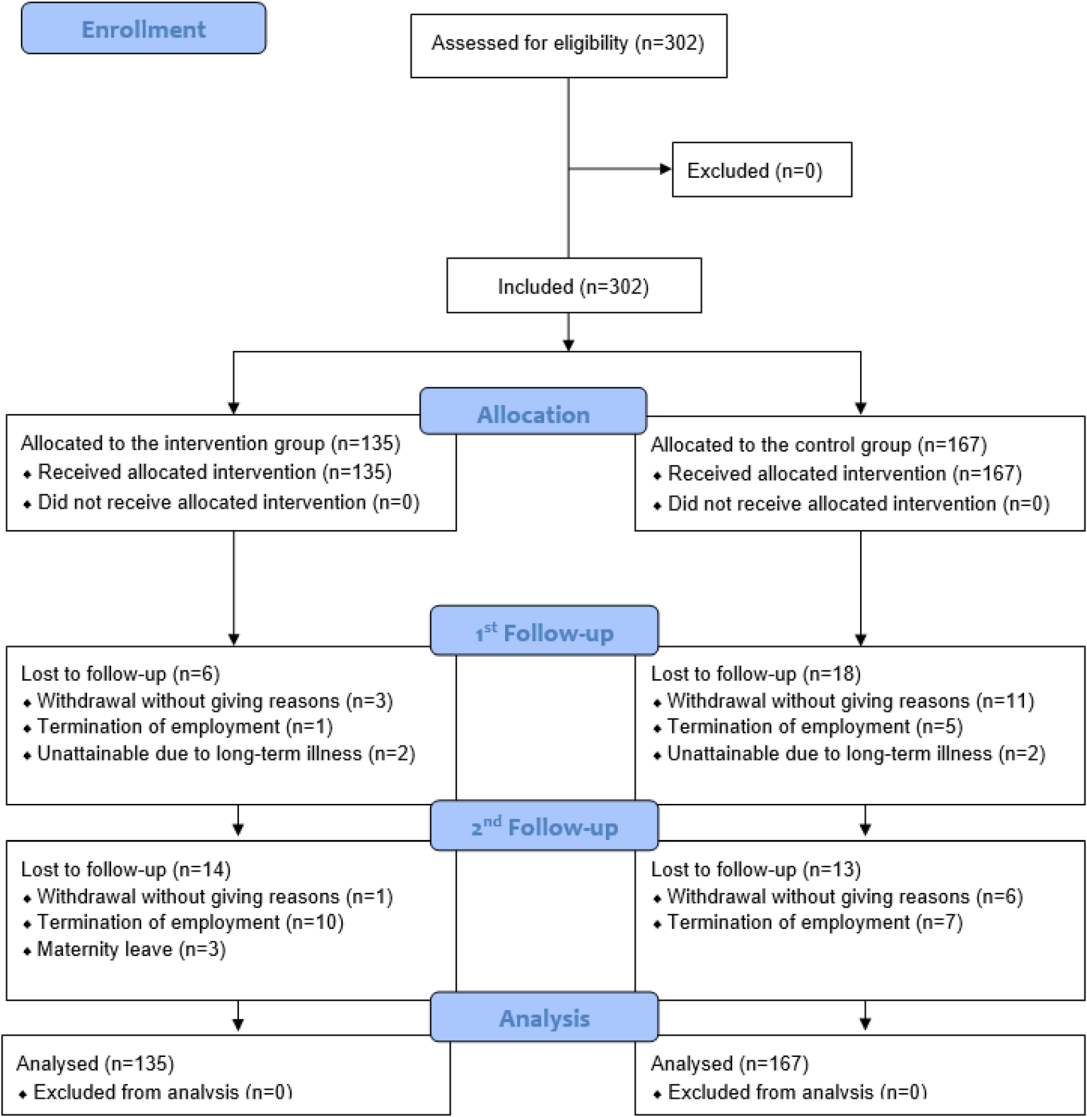
Consolidated Standards of Reporting Trials (CONSORT) 2010 flow diagram for T0 (baseline), T1 (after 3 months; 1st follow‐up) and T2 (after 6 months; 2nd follow‐up); multiple imputation (30 imputations) was conducted by applying fully conditional specification to counterbalance missing data

The participant flow—including reasons for losses to follow‐up—according to the Consolidated Standards of Reporting Trials (CONSORT) 2010^24^ is provided in Figure 1. Overall, 51 (16.9%) HCWs were lost to follow‐up. By most of them (41.2%), the reason for withdrawal was not provided. From 135 participants in the IG, 20 HCWs were lost to follow‐up (14.8%) and from 167 participants in the CG, 31 HCWs were lost to follow‐up (18.6%).

### 3.3 Baseline data

The demographic and clinical characteristics of the participants were similar in both groups (Table 1). The IG (*n* = 135) and CG (*n* = 167) comprised 85.9% and 82.6% female participants, respectively. Participants were on average 36.4 ± 13.5 years old in the IG and 41.1 ± 11.7 years old in the CG. Most of the participants (47.4% in the IG and 55.1% in the CG) held a German ‘Realschulabschluss’ (intermediate school‐leaving certificate). The majority of the participants in the IG (51.1%) and CG (58.7%) were nurses. Most of the participants had been in the occupation for more than 20 years in the IG (41.9%) and 1–5 years in the CG (41.5%). The weekly duration of occupational activity was on average 37.4 ± 8.2 h in the IG and 35.6 ± 8.8 h in the CG. 37.0% of participants in the IG and 32.9% of participants in the CG suffered from hay fever. A similar share of participants of the IG (10.4%) and the CG (10.8%) had asthma. 23.7% and 17.4% of participants in the IG and CG, respectively, reported to have had an itchy rash that has been coming and going for at least 6 months. Involvement of the skin creases was reported by 50.0% and 51.7% of the participants in the IG and CG, respectively. 31.9% of the participants in the IG and 24.0% of the participants in the CG reported to smoke cigarettes.

**TABLE 1:** Baseline demographic and clinical characteristics of the participants in the intervention group (IG), the control group (CG) and overall

	IG (*n* = 135)	CG (*n* = 167)	All (*n* = 302)
Female gender, *n* (%)	116 (85.9)	138 (82.6)	254 (84.1)
Age in years, mean ± SD	36.4 ± 13.5	41.1 ± 11.7	39.0 ± 12.7
Highest graduation, *n* (%)			
None	2 (1.5)	None	2 (0.7)
German Hauptschulabschluss (basic school‐leaving certificate)	4 (3.0)	8 (4.8)	12 (4.0)
German Realschulabschluss (intermediate school‐leaving certificate)	64 (47.4)	92 (55.1)	156 (51.7)
Specialized A‐levels	32 (23.7)	37 (22.2)	69 (22.8)
A‐levels	33 (24.4)	30 (18.0)	63 (20.9)
Occupational activity, *n* (%)			
Nurse	69 (51.1)	98 (58.7)	167 (55.3)
Auxiliary nurse	18 (13.3)	13 (7.8)	31 (10.3)
Physician assistant	5 (3.7)	22 (13.2)	27 (8.9)
Surgical assistant	25 (18.5)	1 (0.6)	26 (8.6)
Technical sterilization assistant	14 (10.4)	2 (1.2)	16 (5.3)
Care assistant	1 (0.7)	7 (4.2)	8 (1.0)
Geriatric nurse	None	3 (1.8)	3 (1.0)
Anaesthesia assistant	1 (0.7)	None	1 (0.3)
Midwife	2 (1.5)	None	2 (0.7)
Chemical‐technical assistant	None	1 (0.6)	1 (0.3)
Occupational therapist	None	3 (1.8)	3 (1.0)
Physical therapist	None	9 (5.4)	9 (3.0)
Osteopath	None	1 (0.6)	1 (0.3)
Participants per assigned workplace, *n* (%)			
Normal ward	109 (80.7)	142 (85.0)	251 (83.1)
Central sterile supply department	21 (15.6)	1 (0.6)	22 (7.3)
Outpatient clinic	5 (3.7)	3 (1.8)	8 (2.6)
Intensive care unit	None	21 (12.6)	21 (7.0)
Duration of the period of occupational activity, *n* (%)			
<1 year	5 (3.0)	15 (11.1)	20 (6.6)
1–5 years	27 (16.2)	56 (41.5)	83 (27.5)
6–10 years	28 (16.8)	14 (10.4)	42 (13.9)
11–20 years	37 (22.2)	15 (11.1)	52 (17.2)
>20 years	70 (41.9)	35 (25.9)	105 (34.8)
Weekly working hours, mean ± SD	37.4 ± 8.2	35.6 ± 8.8	36.4 ± 8.5
Atopic diathesis, *n* (%)			
Hay fever	50 (37.0)	55 (32.9)	105 (34.8)
Asthma	14 (10.4)	18 (10.8)	32 (10.6)
Itchy rash that has been coming and going for at least 6 months	32 (23.7)	29 (17.4)	61 (20.2)
If itchy rash: skin creases affected^a^	16 (50.0)	15 (51.7)	31 (50.8)
Smoking cigarettes			
Smokers, *n*, %	43 (31.9)	40 (24.0)	83 (27.5)
Cigarettes/day, mean ± SD	11.6 ± 6.5	14.0 ± 5.0	11.9 ± 7.0

Abbreviations: CG, control group; IG, intervention group; SD, standard deviation.

^a^ The *n* mentioned in the preceding line has to be considered for calculating the percentage in this line.

### 3.4 Drop‐out analysis

According to dropout analysis (Table S5), drop‐outs (i.e., participants not completing all measurement occasions) slightly differed from completers (i.e., participants completing all measurement occasions) with respect to some baseline characteristics. On average drop‐outs were younger, applied hand cream less frequently, were less likely to report itchy rash and were less likely to be smokers at baseline. Thus, excluding drop‐outs from analyses could bias the results. Multiple imputations were conducted to estimate missing values based on the available information (e.g., baseline values, relationships between variables in the data set).

### 3.5 Utilization of the intervention concept by participants

A total of 1800 lipid‐containing syndets and 1800 emollients were distributed to the 135 participants of the IG during the study period. The online training course was accessed by 66.6% of the participants of the IG.

### 3.6 Presence of hand eczema

At T0, 3 of 135 (2.2%) participants in the IG and 13 of 167 (5.3%) participants in the CG had acute HE, defined as the presence of (i) vesicles or (ii) erythema score >2 in combination with a score >2 for at least one other clinical sign (papules, scaling, or fissures) based on the OHSI assessment. At T2, no HE cases were present in the IG (*n* = 115) and 18 acute HE cases were present in the CG (*n* = 136). Between T0 and T2, there were no new cases of HE in the IG (*n* = 115) whereas there were 12 (8.8%) new cases of HE in the CG (*n* = 136).

### 3.7 Calculated hand eczema prevalence within healthcare workers in Germany

For calculating the point prevalence of HE within the target population of HCWs in Germany, Bayesian multilevel regression with poststratification (MRP) was deployed (Appendix S6). MRP revealed a point prevalence for HE in HCWs within Germany of 5.8% at T0 based on the dermatologically assessed data, which was slightly larger than the estimate of 4.6% based on self‐reported data (Table 2). Self‐reported data on HE did merely correlate in a moderate way with the dermatologically assessed data (*r* = 0.31).

**TABLE 2:** Self‐reported and dermatologically assessed point prevalence of hand eczema at baseline. Raw and adjusted (for gender and age) prevalence rates are displayed (*n* = 302)

Data source	Raw prevalence	Adjusted prevalence
Questionnaire^a^	4.3%	4.6%
Osnabrueck Hand Eczema Severity Index (OHSI)^b^	5.3%	5.8%

^a^ Question about whether the participants who ever had hand eczema have it right now (at baseline).

^b^ Hand eczema was defined as (i) vesicles or (ii) erythema scoring >2 combined with ≥1 of the symptoms papules, scaling scoring >2 or fissures scoring >2 at the clinical examination with OHSI assessment (modified from Reich et al.^21^); results are based on pooled data of 30 imputations.

### 3.8 Factors associated with hand eczema

Logistic regression predicting HE (OHSI‐based assessment) at T2 in the control group revealed that only an atopic skin diathesis (i.e., itchy rash with skin creases affected) seems to be a relevant predicting factor for higher odds of developing HE (Table 3).

**TABLE 3:** Logistic regression predicting hand eczema (OHSI‐based assessment) at T2 in the control group (*n* = 160)

	*b*	*SE*	*t*	*df*	*p*	95% CI—lower bound	95% CI—upper bound
Intercept	−0.55	1.33	−0.41	112.88	0.681	−3.19	2.09
Age	−0.01	0.02	−0.43	124.72	0.670	−0.06	0.04
Gender	−0.65	0.73	−0.89	123.38	0.375	−2.09	0.79
Frequency of hand washing at work	−0.35	0.33	−1.05	141.45	0.294	−0.99	0.30
Frequency of hand washing at home	−0.35	0.36	−0.95	137.24	0.345	−1.07	0.38
Atopy: skin creases affected	1.87	0.71	2.64	134.64	0.009	0.47	3.27
Atopy: hay fever	0.51	0.61	0.83	124.56	0.410	−0.71	1.72
Atopy: asthma	−0.26	0.94	−0.28	131.77	0.781	−2.13	1.60
Frequency of applying hand cream at work	−0.06	0.19	−0.32	125.78	0.753	−0.43	0.31
Frequency of applying hand cream at home	0.34	0.19	1.82	113.96	0.072	−0.03	0.72

*Note*: All predictors were assessed at baseline. Gender: male = 0, female = 1; atopy variables: no = 0, yes = 1; *b* = regression coefficients on the log scale; results are based on pooled data of 30 imputations.

### 3.9 Clinical signs assessed by the OHSI

**FIGURE 2:**
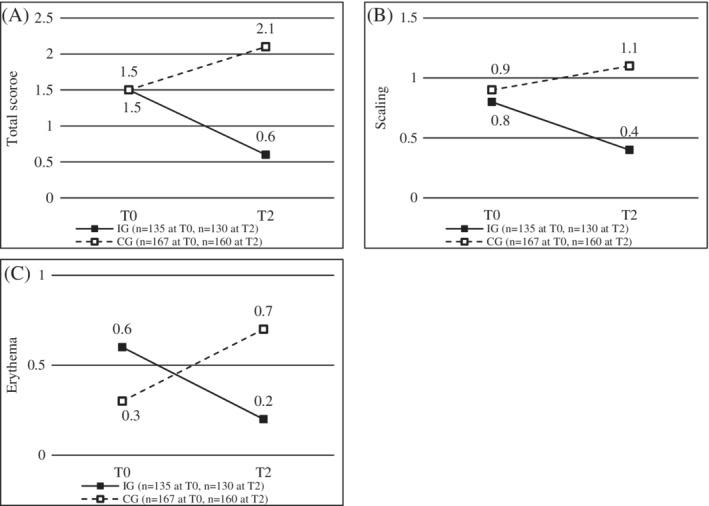
Osnabrueck Hand Eczema Severity Index (OHSI) at baseline (T0) and after 6 months (T2) for the intervention group (IG, *n* = 135 at T0, *n* = 130 at T2) and the control group (CG, *n* = 167 at T0, *n* = 160 at T2): (A) total score, (B) scaling and (C) erythema; pooled data of 30 imputations are displayed as mean values

At T0, most of the participants showed ≥1 clinical sign of hand eczema assessed with the OHSI (overall: 65.2% of 302; IG: 71.9% of 135; CG: 59.9% of 167). Scaling was noted most frequently. At T2, skin changes were present in 41.7% of the participants in the IG (*n* = 115) and 65.4% of the participants in the CG (*n* = 136). Results concerning the OHSI are displayed in Figure 2. At T0, both groups provided equal or nearly equal values. Regarding the total score which was similar for both groups at T0, there was a statistically significant difference between IG and CG at T2 with lower values in the IG than in the CG (*b* = −1.44, *p* < 0.001) and a statistically significant difference between IG and CG with respect to improvement within the observation period (*b* = −0.24, *p* < 0.001) with decrease of the values in the IG and increase of the values in the CG. For scaling a statistically significant difference was observed between IG and CG at T2 with lower values in the IG than in the CG (*b* = −0.74, *p* < 0.001) as well as a statistically significant difference between IG and CG with regard to improvement within the observation period (*b* = −0.10, *p* < 0.001) with decrease in the IG and an increase in the CG. In terms of erythema, there was a statistically significant difference between IG and CG at T2 with lower values in the IG than in the CG (*b* = −0.47, *p* < 0.001) as well as a statistically significant difference between IG and CG with respect to improvement within the observation period (*b* = −0.12, *p* < 0.001) with an improvement of the values in the IG and a deterioration of the values in the CG. For vesicles, infiltration, (but not papules and fissures) differences between IG and CG were also found. However, the magnitude was smaller. The respective results are displayed in Figure S1.

### 3.10 Skin care behaviour

**FIGURE 3:**
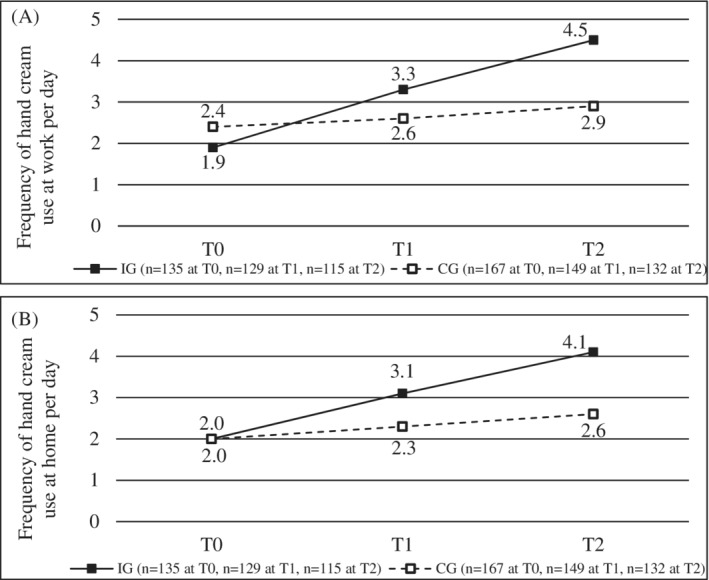
Frequency of emollient use per day at baseline (T0), after 3 months (T1) and after 6 months (T2) in the intervention group (IG, *n* = 135 at T0, *n* = 129 at T1, *n* = 115 at T2) and control group (CG, *n* = 167 at T0, *n* = 149 at T1, *n* = 132 at T2) (A) at work and (B) at home; pooled data of 30 imputations are displayed as mean values

Results on the self‐reported daily frequency of emollient use at work and at home are presented in Figure 3. At T0, both groups provided equal or nearly equal values. Regarding self‐reported daily frequency of emollient use at work, there was a statistically significant difference between IG and CG at T2 with higher frequency in the IG than in the CG (*b* = 1.73, *p* < 0.001) and a statistically significant difference between IG and CG with respect to improvement within the observation period with stronger improvement of the values in the IG compared to the CG (*b* = 0.35, *p* < 0.001). For the self‐reported daily frequency of emollient use at home there was a statistically significant difference between IG and CG at T2 with higher values in the IG than in the CG (*b* = 1.62, *p* < 0.001) and a statistically significant difference between IG and CG with regard to improvement within the observation period with stronger improvement of the values in the IG compared to the CG (*b* = 0.28, *p* < 0.001).

## 4 DISCUSSION

With this interventional trial in HCWs, we were able to show that a two‐part intervention consisting of free access to an adequate hand cleansing product and emollient accompanied by free access to an online‐based health education was effective not only in the prevention of incident hand eczema but also in improving the skin condition of the hands. A strength of this study is that regardless of the pandemic circumstances, a dermatological assessment of the skin condition was conducted which provided objective data on the presence of hand eczema and type of skin lesions. Differences in baseline characteristics of the participants in IG and CG were small or negligible. Since drop‐out rates in studies with similar cohorts of participants often are high,^14^, ^15^, ^16^, ^25^ the rather low drop‐out rate of 16.9% is another strength of this work. Due to the slight differences in baseline characteristics of drop‐outs and completers, multiple imputation was conducted to estimate missing values based on the available information (e.g., baseline values, relationships between variables in the data set). As can be seen in the listing of the assigned workplaces in the baseline characteristics (Table 1), the vast majority of the participants of both groups was recruited on normal wards. It is plausible to assume that the exposure and risk in terms of developing HE were similar within the different departments of the clinics, since the COVID‐19‐associated hygiene measures were the same. Overestimation of intervention effects is thus unlikely. Due to hygiene regulations, we were not allowed to include and examine HCWs working on exclusive COVID‐19 wards; however, due to the exponentially rising COVID‐19‐incidence in the observation period, on most wards, there were COVID‐19 diseased patients, at least intermittently. Due to the limited numbers of participants in the various occupational groups, comparison of results was not possible and could be of interest in future studies (e.g., nurses vs. auxiliary nurses).

Bregnhøj et al.^26^ evaluated the validity of self‐reporting of hand eczema in hairdressing apprentices and found that there was good agreement between self‐reporting of hand eczema and clinical examination. However, in the present study, analysis of dermatologically assessed (clinical examination, OHSI) and self‐reported data on current HE showed that both measurement methods only correlate moderately. This might be related to the definition of HE in this study, but still corroborates the assertion that objective HE data (i.e., clinical examinations) should be preferred over subjective data (i.e., self‐reporting).

Compared to the average point prevalence of HE in the general population of approximately 4%,^27^ the calculated point prevalence (adjusted for gender and age) of HE in HCWs ascertained by clinical examination in our cohort from Germany of 5.8% at T0 is higher indicating that HCWs constitute a high‐risk group for the development of occupational HE.^28^ In previous studies on HCWs, the point prevalence is considerably higher with around 20%^2^, ^29^; this difference may be attributed to the disparity between self‐reported and clinical examination‐based studies. This assumption is also supported by the fact that in our study self‐reported data on hand eczema did merely correlate in a moderate way with the dermatologically assessed data, as was also shown in former studies.^30^

It should be noted that within the study cohort, the prevalence of clinical signs associated with HE was high at T0, whereby scaling as clinical sign of dry skin was most commonly observed. This coincides with current data by Lan et al.^11^ from China, who report on xerosis cutis being a frequent adverse skin condition in HCWs during the COVID‐19 pandemic.

In terms of risk factors, atopic skin diathesis increased odds of developing HE in the control group in the present study. This was to be expected as the relation of an atopic skin diathesis and the development of HE is well described.^31^ This finding, however, stresses the need for preventative measures, especially in the group of people affected by an atopic skin diathesis. It is important to note that there were only 18 HE cases in the CG at T2. In a sample with a larger number of HE cases, probably further relevant factors could have been identified. This should be monitored in future studies.

Based on the OHSI total score and the OHSI values for scaling and erythema, there was a statistically significant difference between IG and CG at T2 (which was not the case at T0; values at T2 were better—i.e., lower—in the IG) as well as between IG and CG with respect to improvement over the course of the observation period. The circumstance that there is only an improvement in the IG and even an aggravation of the skin condition in the CG indicates that the intervention was effective in improving existing skin changes on the hands. The lack of statistically significant changes for papules and fissures can be explained by the fact that values for these signs were already very low at T0. The statistically significant but descriptively small differences for vesicles and infiltration might be explained by the large sample and are probably not of a practical relevance. The overall low OHSI scores at T0 suggest that primarily participants with mild or no skin changes participated.

In the present study, daily frequency of emollient use at work and at home was considered an indicator for skin care behaviour. For both, statistically significant differences between IG and CG at T2 as well as between IG and CG with respect to the improvement over the course of the observation period were observed. The considerable rise in self‐reported frequency of emollient use at work and at home in the IG might have contributed to the improved skin condition at follow‐up. Marginal increases of self‐reported frequencies of emollient application in the CG might be related to social desirability of the answers and attention towards skin care raised by participation in the study. Ibler et al.^16^ conducted a randomized clinical trial in which skin care education and individual counselling were compared to treatment as usual in healthcare workers with HE (secondary prevention) and concluded that—other than in the present study, which has shown that improvements are possible—the two groups did not differ significantly regarding use of emollients at work even though the programme overall improved severity and quality of life and had a positive effect on self‐evaluated severity of HE. The mentioned differences could result from the fact that Ibler et al.^16^ only included people with HE, leading to an already high use of emollients among the participants. In this study, participants who only had mild skin changes or no skin changes were included with possibly greater potential in motivating people to use emollients who have not used it before.

As limiting factors, it should be mentioned that the study was not observer‐blinded and not randomized. Moreover, it cannot be fully ensured that all provided study products (i) were only used by participants and were not distributed further to friends, family, etc., and (ii) were only used according to their intended application. Another limitation of this work is that no long‐term effects of the intervention were evaluated. The observation period of 6 months is suitable for assessing medium‐term effects. A longer observation period was not feasible under the pandemic circumstances this study was conducted in. However, assessing long‐term effects seems particularly important in consideration of a randomized clinical trial by Graversgaard et al., who found that effects of health education attenuated over time with no long‐term effect on outcomes (in this case: presence and severity of hand eczema, health‐related quality of life, skin protective behaviour, knowledge of skin protection and general improvement/worsening of hand eczema), which points to the implication that health education measures should be repeated regularly.^16^, ^32^

The products used in this study were kindly provided in the sense of *proof‐of‐concept* by the manufacturer free of charge over the whole study period in unlimited amounts. The hand washing oil was scented. Generally, from a dermatological and allergological point of view, unscented products are recommendable, even though the scent of a product might have a positive effect on user acceptance.^33^ As the hand washing oil is a rinse‐off product, it was considered tolerable to have fragrances, also keeping in mind the varying sensitizing potential of different fragrances.^34^ The hand cream contained oat flour, which can be a skin sensitizer. However, reports on this allergen are rare^35^ and none of the participants had to be excluded due to an allergy to this substance; the same applies for fragrances. Also, we did not observe any adverse skin reactions to the products provided.

The implemented online health education based on an asynchronous store‐and‐forward technology combined with information sheets (print‐outs) enabled a flexible and time‐efficient access to the educational contents, as it has already been done in a slightly different manner, for example, by Madan et al.^36^ (online behavioural change programme plus hard copy/magazine) or Mollerup, Harboe and Johansen (user access to a website).^37^ Such an intervention concept seems especially appropriate in HCWs who have a high workload and limitation in time. Further, asynchronous online‐based health education also entails time saving for the educator as many recipients could be reached without the effort of face‐to‐face teaching in smaller groups which would have been particularly difficult during the COVID‐19 pandemic. Of course, in the beginning of the study at T0, each participant of the IG had been seen in person by the investigating dermatologist and at this occasion specifically been motivated to make use of the online health education. It must be mentioned, however, that the online‐based health education measure was only accessed by 66.6% of the participants in the IG, which is a serious disadvantage of this technology as it does not automatically provide any option to ensure that the content is consumed as desired. As a further development, it would be imaginable to monitor participation and contact participants who did not use the offered measure (closed system in which activity can be monitored). This way it would be possible to monitor missing utilization at every measurement occasion, assess reasons for non‐utilization and improve the concept accordingly. For complex interventions, intervention effects cannot be ascribed to specific parts of the intervention but only to the concept as a whole. In future studies, the study design could be modified in terms of adding two more intervention groups (one group only getting online‐based health education and one group only getting the products). This would enable examining whether a specific component of the intervention is particularly effective or whether the two‐part intervention concept only works as a whole. Moreover, hands‐on teaching with practical exercises might be more effective in improving behaviour. Prospectively, the described intervention could be used as is or could—when the pandemic conditions again facilitate face‐to‐face methods of health education—partly be adapted and integrated into these educational concepts also in order to increase participation. Particularly, healthcare trainees may benefit from such interventions in order to prevent onset of HE at an early career stage.

## 5 CONCLUSION

The results of this study highlight that realization of adequate infection control concomitant with appropriate hygiene measures should go hand in hand with the implementation of adapting skin care regimes in order to efficiently promote skin health in HCWs. This may prevent individual suffering and impaired quality of life caused by HE as well as minimize costs of illness for social insurance systems and employers. Additionally, the manpower of HCWs—who are urgently needed—is preserved, which seems especially relevant in light of the ongoing COVID‐19 pandemic.^38^, ^39^, ^40^, ^41^ The present study contributes to addressing the need of detailed, comprehensive and purposeful interventional studies with the aim of preventing occupational dermatoses in HCWs, which has frequently been demanded by experts.^13^, ^42^, ^43^, ^44^ It is conceivable that the intervention might also be effective in various other skin hazardous professions, even beyond the human service sector.

## AUTHOR CONTRIBUTIONS

**Cara Symanzik:** conceptualization (lead); data curation (lead); formal analysis (lead); investigation (lead); methodology (lead); project administration (lead); visualization (lead); writing – original draft preparation (lead); writing – review & editing (equal); **Lukasz Stasielowicz:** data curation (equal); formal analysis (equal); software (lead); writing – review & editing (equal); **Richard Brans:** conceptualization (equal); methodology (equal); writing – review & editing (lead); **Christoph Skudlik:** conceptualization (supporting); funding acquisition (lead); methodology (supporting); writing – review & editing (equal); **Swen M. John:** conceptualization (lead); funding acquisition (lead); methodology (lead); project administration (lead); resources (lead); supervision (lead); writing – review & editing (lead).

## CONFLICT OF INTEREST

The authors declare no conflict of interest.

## Supporting information


**Appendix S1** Supporting Information

**Figure S1** Osnabrueck Hand Eczema Severity Index (OHSI) at baseline (T0) and after 6 months (T2) for the intervention group (IG, *n* = 135 at T0, *n* = 130 at T2) and the control group (CG, *n* = 167 at T0, *n* = 160 at T2): (a) papules, (b) vesicles, (c) infiltration, (d) fissures; pooled data of 30 imputations are displayed as mean value.

## Data Availability

The data that support the findings of this study are available from the corresponding author upon reasonable request.
