# Supplementary material for: Prevention of occupational hand eczema in healthcare workers during the COVID‐19 pandemic: A controlled intervention study
Source: Contact Dermatitis. 2022 Aug 30:10.1111/cod.14206. Online ahead of print. doi: 10.1111/cod.14206 (PMC9538141; doi:10.1111/cod.14206)
Supplement: Supplementary file 1 — Appendix S1 Supporting Information [file COD-9999-0-s001.docx]

## Appendix S1

Table S1: Subject areas and learning objectives of the 35-minute-long health pedagogical online training programme (indicative target: participants implement a considerate and careful skin cleansing and skin care behaviour within their private and occupational surroundings) including detailed targets of the individual training sections

| **Targets of the health pedagogical online training in different training sections** |
| --- |
| **Welcome & start** |
| *Participants…*  … get a proper overview about central training objectives.  … assess their individual occupational skin strain.  … assess their individual risk for developing occupational (hand) dermatoses / HE. |
| **Introduction to the topic** |
| *Participants…*  … understand that the increase of hygiene measures during the COVID-19 pandemic has led to an increased skin strain and thereby, to an increase of HE within the general population as well as in particular high-risk groups as HCWs. |
| **Particular issues of HE in HCWs** |
| *Participants…*  … know that a particular problem is persistent regarding the relation of HE and nosocomial infections. The risk of colonisation and, as a consequence, infection by methicillin-resistant *Staphylococcus aureus* (MRSA) is particularly high for HCWs who are already suffering from HE, as pathogens are more easily acquired due to the skin barrier dysfunction and can displace the physiological flora. |
| **Need of implementing adequate skin care in hand hygiene measures** |
| *Participants…*  … know that the latest COVID-19-associated hygiene measures are not complete in terms of adequate skin care.  … know that hygiene measures should be complemented by adequate skin care measures. |
| **Structure of the skin and skin barrier function** |
| *Participants…*  … know where the skin barrier (incl. hydro-lipid film) is located. |
| **Risk factors & pathogenesis of HE: effects of frequent handwashing on the skin barrier function** |
| *Participants…*  … understand how frequent handwashing impacts the skin barrier function.  … are able to recognize symptoms of initial HE. |
| **Hand disinfection** |
| *Participants…*  … know the 5 moments for hand hygiene according to the WHO.  … understand that a burning sensation of the skin whilst using hand disinfection is an early warning sign for an impaired epidermal barrier.  … know that disinfecting the hands is less skin irritating than washing the hands.  … understand that hand disinfection should be given priority to handwashing if possible. |
| **Risk factors & pathogenesis: prevalence of HE in men & and women** |
| *Participants…*  … know that HE is more prevalent in women.  … know that the higher prevalence of HE in women is to be traced back to environmental influences. |
| **Risk factors & pathogenesis: threshold for developing HE** |
| *Participants…*  … know that there is an additive effect of repetitive skin strain. The skin barrier function can be exceeded by cumulative skin irritation. |
| **Basics of skincare** |
| *Participants…*  … are able to describe the intended effect of emollients.  … are able to apply emollients properly (e.g. procedure of adequate application).  … know how often and at which points in time emollients should be applied (e.g. ‘three moments of skin cream application’). |
| **Planning of an adequate skin care behavior within the private and occupational setting** |
| *Participants…*  … plan the implementation of an adequate skin care behavior. |
| **Conclusion & farewell** |
| *Participants…*  … know that they should use the provided lipid-containing syndet and emollients over the whole observation period at home and at work.  … know that they can receive the provided products in unlimited amounts. |

HCWs, healthcare workers; HE, hand eczema; WHO, World Health Organization

## Appendix S2

**Links of the list with additional helpful short videos about skin cleansing and skin care**

Video 1: ‘Structure of the skin’ by the employers' liability insurance association

Link: <https://youtu.be/w0sHoZhllAM>

Video 2: ‘Protecting hands when working frequently with water’ by the employers' liability insurance association

Link: <https://youtu.be/u9xHYnTpYbs>

Video 3: ‘Emollient application’ by the employers' liability insurance association

Link: <https://youtu.be/P96Qf1_RwVU>

Video 4: ‘Skin protection in 100 seconds – emollient application’ by the employers' liability insurance association

Link: <https://youtu.be/DHrzeIiBTWU>

Note: As the study was conducted in Germany, those videos are in German language.

## Appendix S3

Table S2: Ingredients of the provided lipid-containing syndet and emollient according to the International Nomenclature of Cosmetic Ingredients (INCI)

| **Lipid-containing syndet** |  | **Emollient** |
| --- | --- | --- |
| **Ingredients (INCI)**† |  | **Ingredients (INCI)**† |
| Glycine Soja Oil |  | Aqua |
| Laureth-4 |  | Glycerin |
| MIPA-Laureth Sulfate |  | Paraffinum Liquidum |
| Ricinus Communis Seed Oil |  | Cetyl Alcohol |
| Poloxamer 101 |  | Glyceryl Stearate |
| Parfum |  | Stearyl Alcohol |
| Aqua |  | Hydrogenated Coco-Glycerides |
| Propylene Glycol |  | Caprylic/Capric Triglyceride |
| Panthenol |  | Octyldodecanol |
| Tocopherol |  | Butyrospermum Parkii Butter |
| Citric Acid |  | Cetyl Palmitate |
| Sodium Citrate |  | Colloidal Oatmeal |
|  |  | PEG-40 Stearate |
|  |  | Glycyrrhiza Inflata Root Extract |
|  |  | Ceramide NP |
|  |  | Menthoxypropanediol |
|  |  | Citric Acid |
|  |  | Sodium Citrate |
|  |  | Decylene Glycol |
|  |  | Phenoxyethanol |
|  |  | Caprylyl Glycol |
|  |  | Benzyl Alcohol |

†as per chronology of the listing on the package; INCI, International Nomenclature of Cosmetic Ingredients

## Appendix S4

With respect to the primary outcome (skin condition) and the secondary outcome (skin care behaviour), multilevel modeling was used. The change over time was analyzed separately for each outcome. The available time points (two for OHSI scores and three for skin care behaviour, respectively) were nested within persons to account for data dependency. The initial time variable was coded on the month scale to reflect the distance between measurement occasions (e.g. 0, 3, and 6 months for outcomes that were assessed on three occasions). Since we were primarily interested in the medium-term effects of the intervention, the time variable was centered at the last measurement occasion by subtracting 6 from the initial time values. This ensures that intercepts indicate differences between CG and IG after six months. Considering that the rate of change of skin care behaviour may be nonlinear (e.g., greater changes in the beginning), a squared time variable was also considered for outcomes that were measured three times. Since it cannot be assumed that the average trajectory of skin condition and skin care behaviour captures the individual trajectories of all people, random effects were included in the models which allows true differences between individuals (random effects for the intercept and time variables). In the current study, random effects for the intercept refer to the differences between participants with respect to skin condition or skin care behaviour at the last measurement occasion (e.g., good vs. bad values after six months). Random effects for the time variable reflect true differences in the change rate over time. Decision whether to include quadratic trends and/or random effects for time variables was made based on model comparisons for the first imputed data set. Specifically, model weights were computed based on the corrected Akaike Information Criterion (AICc). The model with the highest weight was chosen from the set of candidate models (e.g. random effect for the intercept vs random effect for the intercept and random effect for the time variable) and the reported results are based on these final models. This procedure has been applied separately to all outcomes. First, however, models with different fixed effects (e.g. condition, time variable vs condition, time variable, quadratic time variable) were compared using the Maximum Likelihood method for metric outcomes. Next, the described procedure of model comparison with respect to random effects was carried out. In order to choose between models with different random effects the models were estimated by the Restricted Maximum Likelihood method for metric outcomes. All steps are documented (R code and output) at <https://osf.io/tyshu/?view_only=dca9217a95d743b1ac8c2cc3fcacda6c>.

Table S3: Osnabrueck Hand Eczema Severity Index (OHSI) for the IG and the CG at T0 and T2. Displayed data are based on pooled results of all data sets generated by multiple imputation (30 imputations)

|  |  | **T0** | | | | | | |  | **T2** | | | | | | |
| --- | --- | --- | --- | --- | --- | --- | --- | --- | --- | --- | --- | --- | --- | --- | --- | --- |
| **OHSI** |  | **IG** | | |  | **CG** | | |  | **IG** | | |  | **CG** | | |
|  |  | ***M*** | ***SD*** | **N** |  | ***M*** | ***SD*** | **N** |  | ***M*** | ***SD*** | **N** |  | ***M*** | ***SD*** | **N** |
| **Total** |  | 1.5 | 1.5 | 135 |  | 1.5 | 2.1 | 167 |  | 0.6 | 0.8 | 130 |  | 2.1 | 2.5 | 160 |
| **Scaling** |  | 0.8 | 0.9 | 135 |  | 0.9 | 1.0 | 167 |  | 0.4 | 0.6 | 130 |  | 1.1 | 1.1 | 160 |
| **Erythema** |  | 0.6 | 0.8 | 135 |  | 0.3 | 0.8 | 167 |  | 0.2 | 0.5 | 130 |  | 0.7 | 1.0 | 160 |
| **Papules** |  | 0.0 | 0.2 | 135 |  | 0.0 | 0.2 | 167 |  | 0.0 | 0.1 | 130 |  | 0.1 | 0.2 | 160 |
| **Vesicles** |  | 0.0 | 0.2 | 135 |  | 0.0 | 0.2 | 167 |  | 0.0 | 0.1 | 130 |  | 0.1 | 0.3 | 160 |
| **Infiltration** |  | 0.0 | 0.1 | 135 |  | 0.1 | 0.3 | 167 |  | 0.0 | 0.0 | 130 |  | 0.1 | 0.4 | 160 |
| **Fissures** |  | 0.1 | 0.2 | 135 |  | 0.1 | 0.4 | 167 |  | 0.0 | 0.2 | 130 |  | 0.1 | 0.3 | 160 |

CG, control group; IG, intervention group; *M*, mean; N = sample size; OHSI, Osnabrueck Hand Eczema Severity Index; *SD* = standard deviation; T0 = baseline; T2 = after six months

Table S4: Frequency of emollient use among the participants of the IG and the CG in the occupational as well as private surroundings at T0, T1, and T2. Displayed data are based on pooled results of all data sets generated by multiple imputation (30 imputations)

|  |  | **T0** | | | | | | |  | **T1** | | | | | | |  | **T2** | | | | | | |
| --- | --- | --- | --- | --- | --- | --- | --- | --- | --- | --- | --- | --- | --- | --- | --- | --- | --- | --- | --- | --- | --- | --- | --- | --- |
|  |  | **IG** | | |  | **CG** | | |  | **IG** | | |  | **CG** | | |  | **IG** | | |  | **CG** | | |
|  |  | ***M*** | ***SD*** | **N** |  | ***M*** | ***SD*** | **N** |  | ***M*** | ***SD*** | **N** |  | ***M*** | ***SD*** | **N** |  | ***M*** | ***SD*** | **N** |  | ***M*** | ***SD*** | **N** |
| **occupational†** |  | 1.9 | 1.8 | 135 |  | 2.4 | 2.2 | 167 |  | 3.3 | 1.6 | 129 |  | 2.6 | 1.8 | 149 |  | 4.5 | 5.7 | 115 |  | 2.9 | 2.4 | 132 |
| **private‡** |  | 2.0 | 1.7 | 135 |  | 2.0 | 1.8 | 167 |  | 3.1 | 1.6 | 129 |  | 2.3 | 1.8 | 149 |  | 4.1 | 4.1 | 115 |  | 2.6 | 2.1 | 131 |

† frequency of emollient use in the occupational setting per day; ‡ = frequency of emollient use in the private setting per day; CG, control group; IG, intervention group; *M* = mean; N = sample size; *SD* = standard deviation; T0 = baseline; T1 = after three months; T2 = after six months

## Appendix S5

Table S5: Dropout analysis. Baseline demographic and clinical characteristics of the participants, who completed all three measurement occasions (completers) and participants who did not participate in all measurement occasions (drop-outs).

|  | **Completers (n=251)** | **Drop-outs (n=51)** |
| --- | --- | --- |
| **Age in years, mean (SD)** | 40.1 (12.7) | 33.6 (11.3) |
| **Weekly working hours, mean (SD)** | 36.4 (8.3) | 36.5 (9.9) |
| **OHSI score, mean (SD)** | 1.5 (1.8) | 1.5 (2.2) |
| **Cream frequency (job), mean (SD)** | 2.2 (2.1) | 1.8 (1.6) |
| **Cream frequency (home), mean (SD)** | 2.1 (1.9) | 1.6 (1.3) |
| **Condition**, n (%) |  |  |
| CG | 136 (54.2) | 31 (60.8) |
| IG | 115 (45.8) | 20 (39.2) |
| **Gender**, n (%) |  |  |
| Male | 39 (15.5) | 9 (17.6) |
| Female | 212 (84.5) | 42 (82.4) |
| **Itchy rash, n (%)** |  |  |
| Don’t know | 5 (2.0) | 0 (0) |
| No | 190 (75.7) | 46 (90.2) |
| Yes | 56 (22.3) | 5 (9.8) |
| **If itchy rash: Skin creases affected, n (%)**† |  |  |
| Don’t know | 6 (10.9) | 0 (0) |
| No | 22 (40.0) | 1 (20.0) |
| Yes | 27 (49.1) | 4 (80.0) |
| **Smoking cigarettes, n (%)** |  |  |
| No | 179 (71.3) | 40 (78.4) |
| Yes | 72 (28.7) | 11 (21.6) |

† the n mentioned in the preceding line has to be considered for calculating the percentage for this variable; CG, control group; IG, intervention group; OHSI, Osnabrueck Hand Eczema Severity Index SD, standard deviation

## Appendix S6

For calculating the point prevalence of HE within the target population of HCWs in Germany, Bayesian multilevel regression with poststratification (MRP) was deployed. First, a multilevel logistic regression model was applied to the sample data, with HE as outcome, gender as fixed effect and age group (under 30; 30 or older but younger than 50; 50 or older) as random effect. Next, post-stratification was conducted. First, population-level data (census) were used to obtain the combined distribution of age and gender in the health sector: https://www-genesis.destatis.de/genesis//online?operation=table&code=23621-0005&bypass=true&levelindex=0&levelid=1628606972810#abreadcrumb (Table 23621-0005, data from 2019). The population weights were then applied to the results of the logistic regression in order to estimate HE prevalence of HCWs in Germany. All steps are documented (R code and output) at <https://osf.io/tyshu/?view_only=dca9217a95d743b1ac8c2cc3fcacda6c>.
