# Supplementary material for: Prevention of occupational hand eczema in healthcare workers during the COVID‐19 pandemic: A controlled intervention study
Source: Contact Dermatitis. 2022 Aug 30:10.1111/cod.14206. Online ahead of print. doi: 10.1111/cod.14206 (PMC9538141; doi:10.1111/cod.14206)
Supplement: Supplementary file 2 — Figure S1 Osnabrueck Hand Eczema Severity Index (OHSI) at baseline (T0) and after 6 months (T2) for the intervention group (IG, n = 135 at T0, n = 130 at T2) and the control group (CG, n = 167 at T0, n = 160 at T2): (a) papules, (b) vesicles, (c) infiltration, (d) fissures; pooled data of 30 imputations are displayed as mean value. [file COD-9999-0-s002.docx]

Figure S1: Osnabrueck Hand Eczema Severity Index (OHSI) at baseline (T0) and after six months (T2) for the intervention group (IG, n=135 at T0, n=130 at T2) and the control group (CG, n=167 at T0, n=160 at T2): a) papules, b) vesicles, c) infiltration, d) fissures; pooled data of 30 imputations are displayed as mean value
